# HGF-MET as a breast cancer biomarker

**DOI:** 10.18632/aging.100735

**Published:** 2015-03-30

**Authors:** Shuying Liu

**Affiliations:** Departments of Breast Medical Oncology and Systems Biology, The University of Texas MD Anderson Cancer Center, Houston, TX 77030, USA

Although targeting MET has yielded promising results in preclinical studies, few clinical trials of MET inhibitors have demonstrated the expected therapeutic benefits. This inconsistency raises the possibilities that there are different subsets of MET/HGF-aberrant breast cancer with different responses to MET/HGF-targeted therapies and that MET inhibitors benefit only a particular subgroup of patients. To fully realize the benefits of MET inhibitors, we must clarify the patient population that will benefit from them. Therefore, it is crucial to identify biomarkers that will optimize the use of MET inhibitors in individual breast cancer patients.

*MET aberrations*. MET overexpression with or without amplification has been reported in many cancers [1]. MET protein expression and its phosphorylation were aberrantly upregulated in around 70% and 48% breast cancers, respectively, which independently predict poor outcome [2]. The MET signaling also can be aberrantly activated as a consequence of *MET* mutation or functional single nucleotide polymorphisms (SNPs). Several *MET* gene mutations have been reported in various cancers, but oncogenic *MET* mutations occur spontaneously in only 2-3% [1]. Mutation in the tyrosine kinase domain renders the enzyme constitutively active, while mutation in the juxtamembrane domain reduces MET degradation. *MET* sequence changes occur in 9% of patients with breast cancer. However, these appear to represent SNPs rather than somatic mutations. The sequence changes were associated with higher metastatic burden and high-grade histology [3]. A recent study demonstrated that *MET* is functionally altered by an uncommon germline SNP, *MET*-T1010I, which is present twice as frequently in patients with metastatic breast cancer as in the general population. *MET-*T1010I transforms mammary epithelial cells and drives tumor formation and invasion in human *HGF* transgenic mice [4], suggesting that it potentially alters tumor pathophysiology and response to MET-targeted therapies. Therefore, *MET-T1010I* should be considered a potential biomarker when implementing clinical trials of MET-targeted agents.

*Hepatocyte growth factor (HGF)/scatter factor upregulation*. Not only the MET receptor but also its ligand HGF drives tumor formation, metastasis, and drug resistance [1]. We recently established a mouse model system in which the host mice express human HGF at varying levels and the xenografts express human MET receptor of varying status. The models faithfully mimic patients with different HGF levels and different MET receptor status in their breast cancer. Using this model, we found that MCF-10A cells transformed with aberrant MET formed tumors in the mice with transgenic human *HGF* but not in the negative litters. Comparing mutant MET, wild type MET is more stringently dependent on its natural ligand HGF. These data suggest that not only MET status affects cell behavior but also level of its ligand HGF in the tumor microenvironment plays a key role in determining the functional outcomes of MET aberrations. Indeed, overexpression of HGF has been demonstrated in breast cancer, and HGF levels are increased in the serum of patients with breast cancer. Both primary and metastatic tumor cells (autocrine mechanism) and stromal cells (paracrine mechanism) secrete high levels of HGF and aberrantly induce ligand-dependent MET signaling. High HGF level correlates with poor prognosis in breast cancer [1]. Hypoxic conditions stimulate production of both MET receptor and HGF, rendering tumor cells more sensitive to HGF stimulation in the invasion process [5]. Therefore, HGF level also should be considered a potential biomarker when testing MET-targeted agents.

*Crosstalk between MET and other pathways.* Accumulating evidence suggests that MET plays a key role in resistance to targeted therapies for cancer through crosstalk between MET and other pathways, such as the EGFR family [6]. Inhibition of either MET or EGFR was insufficient to fully block signaling in gefitinib-resistant cell lines, whereas the combination completely inhibited signaling. Indeed, combined targeting of MET with onartuzumab and EGFR with erlotinib in a clinical trial prolonged progression-free survival and overall survival in patients with lung cancer expressing high levels of MET [7]. However, the mechanisms of acquired resistance to MET inhibition remain little known. Crosstalk with other oncogenic pathways might induce acquired resistance to MET inhibition. We demonstrated that concurrent aberration of *MET* and *PIK3CA* greatly increased in breast cancer. Our unpublished data show that concurrent aberration of MET and PI3K significantly increased cell proliferation and invasion *in vitro* and in mice with similar human HGF levels. Targeting both MET and PI3K yielded greater inhibitory efficacy than targeting either agent alone, suggesting that response to MET-targeted therapy in breast cancer is dependent on an aberrant MET-HGF/PI3K axis.

Taken together, these findings indicate that, to optimize the use of therapies targeting MET signaling and improve treatment efficacy in individual breast cancer patients, MET status, HGF level, and activation of the MET-HGF/PI3K and EGFR-MET axes should be considered as potential biomarkers when implementing clinical trials of MET-targeted agents.
